# Prenatal caloric restriction alters lipid metabolism but not hepatic *Fasn* gene expression and methylation profiles in rats

**DOI:** 10.1186/s12863-017-0544-0

**Published:** 2017-08-15

**Authors:** Joanna Nowacka-Woszuk, Zofia E. Madeja, Agata Chmurzynska

**Affiliations:** 1 Department of Genetics and Animal Breeding, Poznań University of Life Sciences, Wolynska 33, 60-637 Poznan, Poland; 2 Department of Human Nutrition and Hygiene, Poznań University of Life Sciences, Wojska Polskiego 31, 60-624 Poznan, Poland

**Keywords:** Nutrigenomics, Calorie-restricted diet, Rat, Liver, *Fasn*, Gene expression, DNA methylation, Lipid profile

## Abstract

**Background:**

Undernutrition is an increasingly common problem. Insufficient calorie intake and nutrient deficiencies during pregnancy may have an impact not only on the mother, but may also alter metabolism in the infant. In this study, we have applied a calorie-restricted diet during gestation and examined its effect on hepatic *Fasn* mRNA and DNA methylation profiles in rats and their female progeny. The body composition and blood lipid profiles were also evaluated in both generations.

**Results:**

The results showed that the investigated diet regimen exerted a greater effect on the dams than on the offspring. We found that, in the calorie-restricted group, the transcript level of the *Fasn* gene in the liver increased in the mothers, while in the progeny it was only slightly enhanced. The implemented diet altered lipid profile in the dams by decreasing total cholesterol, HDL, and TG levels. An increase in LDL was noted in the offspring. No change in DNA methylation profile was observed in response to the calorie-restricted diet.

**Conclusions:**

Calorie restriction during pregnancy modified the hepatic *Fasn* mRNA transcript level and altered the blood cholesterol concentrations in dams, but there were no such effects in their four-week-old offspring. The examined dietary regimen had no effect on DNA methylation of the *Fasn* 5′-flanking region in the rat liver.

**Electronic supplementary material:**

The online version of this article (doi:10.1186/s12863-017-0544-0) contains supplementary material, which is available to authorized users.

## Background

Human obesity is one of the major diseases of civilization and its pathogenesis has, to a great extent, a polygenic background. For this reason, the search for candidate genes crucial to its etiology is a difficult task which to date has given inconsistent results. Additionally, several environmental factors contribute to overweight or/and obesity. It is worth mentioning that only a small percentage of obesity cases are caused by mutations in single genes, such as *leptin (LEP)*, *leptin receptor* (*LEPR)*, and *melanocortin 4 receptor* (*MC4R)* [1]. Thus, extensive research has been undertaken into the etiology of obesity and its consequences. On the other hand, although almost 795 million people worldwide suffer from hunger, studies of the effects of undernutrition on various aspects of human life are still scarce. In developing countries, more than 120 million women of child bearing age were underweight in 2003 (http://www.worldhunger.org). The quantitative or qualitative undernutrition of women of this age not only affects their health, but also influences fetal development. The children of undernourished mothers are more likely to have low birth weight. In 2013 alone, almost 22 million infants were affected by these problems at birth (http://www.worldhunger.org). The effects of maternal undernutrition, calorie restriction, and deficiencies in macroelements and microelements have recently been studied in human and rodent models [2–4].

The functioning of the adult organism is strongly dependent on factors that affect it during prenatal development. This phenomenon is called “*fetal programming*”. The maternal diet during pregnancy may exert long-term effects on gene expression levels in the progeny through epigenetic mechanisms such as DNA methylation [5]. Undernutrition of pregnant females may predispose their offspring to health problems in the adulthood. Insufficient nutrient availability can lead to intrauterine growth retardation syndrome (IUGR) resulting in low birth weight, changes in blood pressure, and heart disease in adulthood [6]. The impact of diet type during pregnancy not only directly influences offspring, but can also be transmitted to later generations. The study of Hoile [7] attempted to unravel the transgenerational impact of a low-protein diet on a global hepatic transcription profile in rats, and revealed that the number of differently expressed genes varied from 1680 in F1 to 2062 in F3. However, only about 100 genes showed different expression in all three generations. This indicates that only a small number of genes retain transgenerational transmission of the expression profile. It has also been shown that insufficient calorie intake during the preconceptional or gestation period in female rats impairs lipid metabolism, altering the mRNA level of key genes responsible for lipid homeostasis or adipogenesis, such as *Pparg* (*peroxisome proliferator-activated receptor gamma*), *Ppara* ﻿﻿(﻿*peroxisome proliferator-activated receptor alpha*), *Acaca* (*acetyl-CoA carboxylase alpha*), *Fasn* (*fatty acid synthase*), and *Insig1* (*insulin induced gene 1*) in adult offspring [8].

Several genes involved in lipid metabolism have been examined for their role in fetal programming, including the main regulators of metabolism, such as *Srebf1 ﻿* (*sterol regulatory element binding transcription factor 1)*, *Ppars*, and *Lxrs* (*liver X receptors*) [9]. One of the major genes responsible for lipid homeostasis that has not yet been tested for its role in the programming of lipid metabolism by prenatal caloric restriction is *Fasn*. This gene is highly expressed in the liver, and the enzyme catalyzes the synthesis of palmitate from acetyl-CoA and malonyl-CoA into long-chain saturated fatty acids [10]. Moreover, the main regulator of cholesterol synthesis, sterol regulatory element binding protein SREBP, can activate *Fasn* gene expression [11]. There have been only a few studies on the dietary regulation of *Fasn* methylation or its expression. It has recently been shown that a high-protein calorie-restricted (40% reduction) diet in adult rats downregulates the hepatic level of *Fasn* mRNA, but had no effect on the level of Fasn protein. Moreover, in rats fed with the restricted diet, the intrahepatic triglyceride concentration decreased relative to the control group [12]. Lomba [13] studied the regulatory region of *Fasn* in adipose tissue and showed that a high-fat diet in adult rats decreased its DNA methylation level; however, there was no correlation of methylation with the expression profile of *Fasn*.

Although the metabolic effects of fetal programming may occur later in life [5], changes in DNA methylation are sensitive to environmental factors, and it is thus reasonable to examine methylation profiles in young animals. The main aim of this study was to verify the hypothesis that restricted calorie intake during rat pregnancy can modulate the methylation profile and expression of the *Fasn* gene in the liver of the progeny, and that these effects may be observed just after weaning. We also examined whether this experimental procedure affected lipid metabolism in pregnant dams or in the four-week-old progeny.

## Methods

### Experimental procedures

This study was undertaken as a part of an extensive research project aimed to analyse the transgenerational inheritance of methylation patterns induced by prenatal caloric restriction. The animal protocol for the entire study was approved by the local ethics committee (approval no. 37/2014). Wistar rats aged 10 weeks were purchased from Charles River Laboratories (Germany). Rats were housed in individual cages on a 12 light–dark cycle (light on from 08:00 to 20:00) at temperature of 20 ± 1 °C. After an acclimatization period, twelve virgin female rats were mated with twelve males. During the mating phase, the animals were allowed to eat ad libitum. Successful mating was confirmed by the presence of a vaginal plug, and the female rats (F0 generation) were assigned either to the control diet (the AIN-93G diet ad libitum) or the caloric restriction diet. The caloric-restriction group (R) was fed 50% of the typical food intake of the control (C) group, with a correction for body mass. After delivery, the litter size was recorded. Three days after delivery, the litters were culled to a maximum of eight pups (F1 generation) to minimize variation in nutrition during the suckling period. Following parturition, all rats were introduced to the AIN-93G diet ad libitum and continued to receive this diet for the remainder of the experimental protocol.

Food intake was monitored every day during pregnancy. The weight of the pregnant dams (F0) was measured weekly using electronic scale. The body composition of four-week-old female rats of the F1 generation was analyzed using a Minispec LF90II (Bruker, Germany).

At the end of the experimental period, the animals were fasted overnight, anesthetized by CO_2_ inhalation, and euthanized by cardiac puncture. Liver samples were taken, immediately frozen in liquid nitrogen and stored at −80 °C for further analysis. Each group (F0 C, F0 R, F1 C, and F1 R) was composed of six female rats.

### Biochemical analyses

Blood samples for the biochemical measurements were allowed to clot at room temperature for 30 min. The serum was stored at −80 °C until analysis. Concentrations of serum total cholesterol, LDL cholesterol, HDL cholesterol, and triglycerides (TG) were analyzed using commercial kits (Thermo Fisher Scientific, Waltham, MA, USA) and standard enzymatic methods with a Konelab 20i fully automated analyzer (Thermo Electron Corporation, Vantaa, Finland).

### Relative transcript level analysis

TriPure reagent (Sigma-Aldrich, St. Louis, MO, USA) was used for hepatic total RNA isolation according to the standard procedure. The quantity and quality of isolates were monitored on a Nanodrop spectrophotometer. For reverse transcription, 2 μg of RNA was used and the reaction was performed with a Transcriptor High Fidelity cDNA Synthesis kit (Roche, Indianapolis, IN, USA). SYBR Green detection system was applied for semiquantitative mRNA transcript level measurements (LightCycler 480 SYBR Green I Master Kit, Roche, Indianapolis, IN, USA). Real-time PCR reactions were conducted on a LightCycler 480 II (Roche, Indianapolis, IN, USA). The PCR primers for the selected fragment of the *Fasn* gene were adopted from Sawano et al. [14]. Primer sequences for the two reference genes (*Hprt, hypoxanthine-guanine phosphoribosyltransferase gene* and *Tbp, TATA box binding protein gene)* were chosen, as described by Nowacka-Woszuk [15]. Each sample was analyzed in duplicate and quantification was performed after normalization of the *Fasn* mRNA level to the transcript level of the reference genes [16].

### Western blot analysis

Total protein content was isolated from liver tissue samples using a RIPA lysis buffer (Sigma-Aldrich, St. Louis, MO, USA). The efficiency of protein isolation was monitored on a Qubit fluorometer using a Protein Assay Kit (Thermo Fischer Scientific, Waltham, MA, USA). The protein samples (25 μg) were separated on 10% SDS PAGE gel by electrophoresis (120 V, 45 min) followed by dry transfer (iBlot, Thermo Fisher Scientific, Waltham, MA, USA) on to nitrocellulose membrane (Life Technologies, Waltham, MA, USA). The membranes were initially blocked for 60 min with 10% nonfat milk in TBST solution, washed with TBST buffer, and then incubated overnight with the primary antibody (diluted 1:5000 in TBST) directed against Fasn protein (rabbit monoclonal antibody, ab128870, Abcam, Cambridge, UK). Next, the membranes were washed 3 times for 10 min in TBST buffer, following incubation for 60 min with the secondary antibody (goat anti-rabbit, ab6721, Abcam, Cambridge, UK) conjugated with horseradish peroxidase. After 3-10 min washes, the signals for Fasn were detected. β-actin served as reference for protein level analyses. For this purpose, the membrane was again incubated with the primary antibody (1:2500 dilution in TBST) directed against β-actin (mouse monoclonal antibody ab8226, Abcam, Cambridge, UK) overnight, following 3-10min washes in TBST and incubation for 60 min with the secondary antibody (goat anti-mouse ab98808, Abcam, Cambridge, UK) conjugated with horseradish peroxidase. Next, 3-10 min washes were performed and the signals for β-actin were recorded. The presence of the investigated proteins was detected by chemiluminescence (ECL Prime Western Blotting Detection Reagent, GE Healthcare, Piscataway, NJ, USA) on a Versa Doc scanner (BioRad, Hercules, CL, USA). Quantification based on band density was established using ImageJ software (https://imagej.nih.gov/ij/).

### DNA methylation analysis

The search for CpG island (CGI) was undertaken in the region of 2 kb at the 5′ end of the *Fasn* gene, which can be potentially involved in transcription regulation (GenBank_NC005109) (http://www.ebi.ac.uk/Tools/seqstats/emboss_cpgplot/). We applied the standard criteria for searching (minimum length > 200 bp, minimum C + G content >50% and observed/expected ratio > 0.6). This allowed us to identify the CGI, which was 233 bp long, overlaps 21 CG dinucleotides, and spans from −735 to −967 bp from the transcription start site, TSS (Additional file 1 Figure S1). The PCR primers for the bisulfite converted sequence (F:5′ TTTGAGTAGTTTGTGTTTTTTTGGT and R:5′ CAAACACCCACCCTTTCTATAAC) were designed using MethPrimer software (http://www.urogene.org/cgi-bin/methprimer/methprimer.cgi) and allowed to amplify the 322 bp fragment.

DNA was isolated from the livers using a 24:25:1 phenol:chloroform:isoamyl alcohol acid mixture (Sigma-Aldrich, St. Louis, MO, USA). For bisulfite conversion (EZ DNA Methylation Kit, Zymo Research, Inc., Irvine, CA, USA), 1 μg of DNA was used, following touchdown PCR as described earlier by Nowacka-Woszuk [14]. The amplicons were cloned into pGEM T-Easy vector (Promega, Madison, WI, USA). Competent *E. coli* bacteria (DH5α strain, Invitrogen, UK) were transformed with recombinant vectors and harvested overnight at 37 °C on agar plates in the presence of ampicillin, X-Gal, and IPTG (A&A Biotechnology, Gdansk, Poland). Plasmid DNA from white colonies was amplified using an Illustra TempliPhi Amplification Kit (GE Healthcare, Piscataway, NJ, USA) for 4–16 h at 30 °C. Next, the samples (minimum eight clones per sample) were sequenced by the Sanger method using a Big Dye Terminator v.1.1 Sequencing kit (Thermo Fisher Scientific, Waltham, MA, USA) on a Genetic Analyzer 3130 system (Applied Biosystems, Foster City, CA, USA). The percentage of methylation in each analyzed CG dinucleotide was calculated using QUMA software (http://quma.cdb.riken.jp/).

### Statistical analysis

The results are presented as medians with interquartile ranges. Between-group differences were tested using the Mann–Whitney test. The Spearman rank–correlation test was used to analyze the relations between all the parameters. Data was analyzed using the Statistica software (Statsoft Inc., Tulsa, OK, USA) and *p* < 0.05 was taken to be statistically significant.

## Results

### Body weight and metabolic profile

**Fig. 1:**
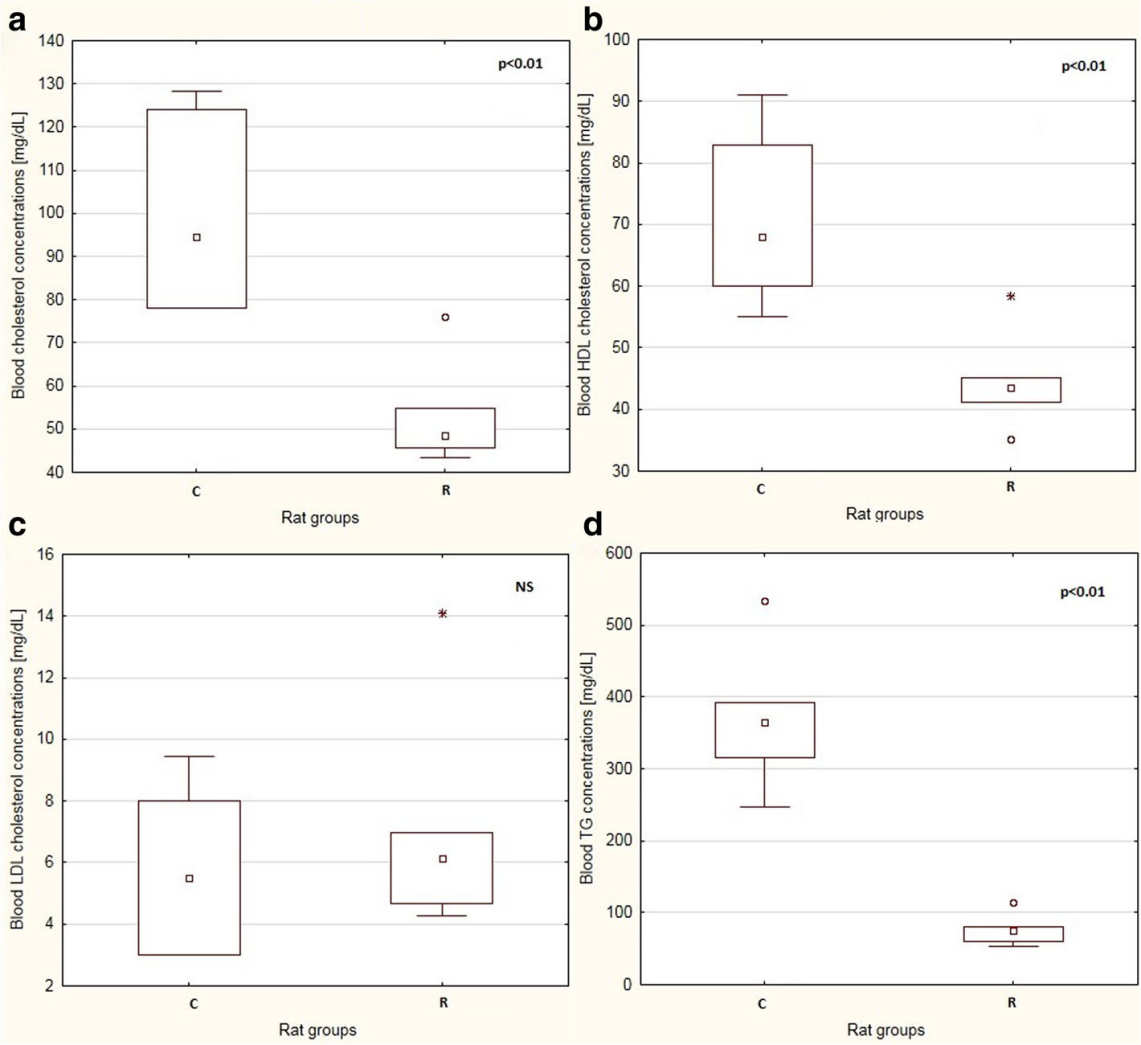
Boxplots showing median values and interquartile ranges of selected blood biomarkers: total cholesterol **a** HDL cholesterol **b**, LDL cholesterol **c**, and TG **d** in the F0 generation. Circles represent outliers that extend more than 1.5 box-lengths from the edge of the box, and stars represent extreme outliers that extend more than three box-lengths; p: level of significance; NS: not significant

**Fig. 2:**
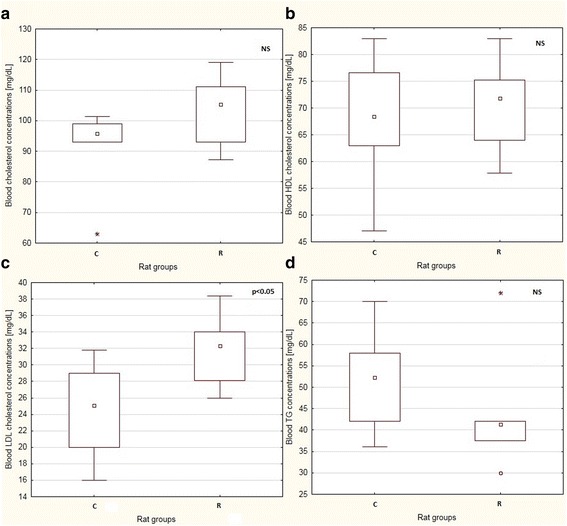
Boxplots showing median values and interquartile ranges of selected blood biomarkers: total cholesterol **a** HDL cholesterol **b**, LDL cholesterol (**c**), and TG **d** in the F1 generation. Ccircles represent outliers that extend more than 1.5 box-lengths from the edge of the box, and the stars represent extreme outliers which extend more than three box-lengths; p: level of significance; NS: non-significant

Caloric restriction led to decreased weight gain during pregnancy. Specifically, there was no statistically significant difference on day 8 between the C and the R groups (246 g ± 34 g and 221 ± 15 g), but on day 15, the difference in the median body mass was 47 g (*p* < 0.01). We observed a consistent tendency towards leaner body composition in the F1 R group than in the F1 C. The median body mass, percentage body fat, and percentage lean body mass in the F1 C group were 65.3 g, 9.2%, and 78.0%, respectively. The same parameters in the F1 R group were 57.9 g, 6.7%, and 79.6%, respectively. However, these differences did not reach statistical significance. The dietary treatment during pregnancy led to a significant decrease in total serum cholesterol, HDL, and TG concentrations (*p* < 0.01 for all associations) in the F0 R group (Fig. 1). However, in the F1 generation, only LDL concentration differed significantly between the dietary groups (Fig. 2): the median value of this parameter increased in the F1 R by over 20%, as compared to the F1 C group.

### Transcript level and protein expression

**Fig. 3:**
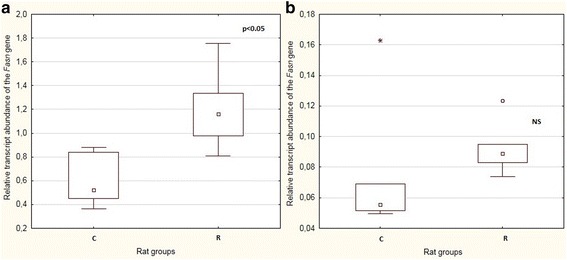
Boxplots showing median values and interquartile ranges of mRNA levels of hepatic *Fasn* in F0 **a** and F1 generation **b**. The circles represent outliers which extend more than 1.5 box-lengths from the edge of the box, and stars represent extreme outliers that extend more than three box-lengths; p: level of significance; NS: not significant

Caloric restriction resulted in an increase in *Fasn* gene transcription in the F0 generation (*p* < 0.05), but this difference was not evident in the F1 animals (Fig. 3). Semiquantitative analysis of the protein content revealed an increase in the Fasn protein in F0 R group compared to the F0 C animals, but these results were not statistically significant (Additional file 2 Figure S2). A similar tendency was found for the F1 generation.

In the F0 generation, the *Fasn* transcript level was inversely correlated with the total serum cholesterol and HDL cholesterol concentrations: *R* = −0.73 and *R* = −0.70, respectively (*p* < 0.05). Such correlations were also found for the Fasn protein and the total serum cholesterol and HDL concentrations: *R* = −0.64 and *R* = −0.64, respectively (*p* < 0.05). In the F1 generation, no correlations were observed between the measured biomarkers and gene transcription or protein levels. However, in the F1 generation, transcript abundance highly correlated with protein levels (*R* = 0.73, *p* < 0.05).

### Methylation analysis

**Fig. 4:**
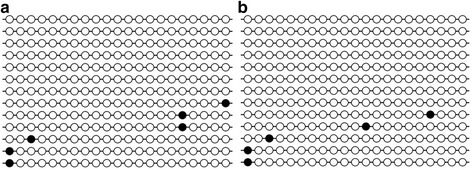
Results of methylation study: a fragment representing 21 CG dinucleotides. Open circles represent unmethylated cytosine and black circles represent methylated cytosine. Each row corresponds to a particular clone sequenced. **a**: sample for an animal from the F0 C group and **b**: sample for an animal from the F0 R group

The methylation analysis of the 5′ flanking region of the *Fasn* gene, which overlapped 21 CG dinucleotides, showed that it was almost completely unmethylated (Fig. 4). The highest average methylation percentages were at CG1 and CG2. The average methylation percentage of the first CG were 12.1% and 7.1% in the F0 C and F0 R groups, respectively, while in the F1 generation, the respective values were 8.0% and 6.7%. Similarly, the average methylation percentage of the second CG was slightly lower in the R groups: 7.4% in the F0 C, 4.2% in the F0 R, 15.5% in the F1 C, and 10.3% in the F1 R. The observed differences were not statistically significant. CGs number 4, 6, 8–11, 13, 15, 16, 19, and 21 were unmethylated in all the analyzed samples in the F0, while CGs number 13, 15, 16, 20 and 21 were unmethylated in the F1. Correlations between the overall methylation level or methylation of the particular cytosine and the measured physiological parameters were not observed. Only methylation of the third CpG was inversely correlated with TG concentrations in the F1 R group (*R* = −0.85, *p* < 0.05). There was no correlation between hepatic DNA methylation and mRNA levels of the *Fasn* gene.

## Discussion

The fetal programming phenomenon has recently been widely investigated. Rat model studies involving calorie-restricted diets during pregnancy can bring new insights into molecular mechanisms of the far-reaching effects of human prenatal undernutrition. In this study, we have used a 50% calorie restriction diet during rat pregnancy and have noted a significant reduction in body weight in pregnant dams accompanied by a tendency towards leaner body composition in their progeny. Similar findings were described by Eleftheriades [17], who also applied a 50% calorie-reduction feed during pregnancy. After delivery, these pups were fed by mothers from a control group, or by dams for which the restricted diet was continued during the whole lactation period. The authors found that the progeny of the prenatal control group were heavier at day 26 of life. Moreover, the postnatal control feeding type of pups from prenatally restricted dams had an increase in abdominal fat content compared to pups starved prenatally and postnatally. This indicates that not only the gestation time, but also the lactation time, is crucial for nutrition programing. A 50% food restriction during pregnancy (resulting in IUGR), followed by a high-sucrose diet after weaning, was employed in the study of Malo [18]. The authors found that one-month old female pups born from IUGR pregnancies exhibited increased cholesterol and HDL cholesterol concentrations, but no effect on body composition with regard to lipid content in different adipose depots was observed. Moreover, a high-fructose diet used after weaning induced lipid profile changes in six-month-old offspring. In our study, where the restricted diet was applied prenatally, the influence on lipid profile was greater in the F0 generation, where we observed a decrease in total cholesterol, HDL cholesterol, and TG concentrations. In the F1 animals, only the LDL cholesterol content was altered. The restrictions of selected nutrients in the maternal diet may also lead to changed lipid metabolism in the progeny. In the study of Fagundes [19], protein-restricted diet was implemented during lactation. This resulted in lower body weight as a consequence of lower visceral and total fat contents. Moreover, blood lipid profile changes (higher LDL cholesterol, lower TG, and lower total cholesterol) were observed. On the other hand, there have been reports showing no effect of prenatal feeding on lipid profile. In the study of Choi [20], a 25% calorie restriction was applied during pregnancy, and no changes were noted in the serum lipid profile. In studies where a protein restricted diet was used during gestation, no effect for lipid profile was found either, but programming of body composition was affected [21].

The restricted diet in our experiment significantly increased the hepatic *Fasn* mRNA level in the F0 generation, while in the F1 animals, only a trend, lacking statistical significance, was observed. Moreover, in the F0 generation, the increase in *Fasn* mRNA and protein levels in the restricted group was correlated with lower serum cholesterol and HDL cholesterol contents. Lowered lipid absorption could possibly lead to activation of the *Srebf1* gene, which is a regulator of the *Fasn* gene. In the F1 R group, we also observed a tendency for the protein content to increase and a strong correlation between mRNA and protein levels in the F1 generation. Similar results were found by Choi [20], where a 25% calorie reduction during pregnancy did not alter the offspring’s hepatic*Fasn* transcription—in contrast to the Fasn protein content, which strongly increased in the progeny of the restriction-treated dams. Unlike our results, Ramírez-López [8] showed that 20% calorie restriction during pregnancy decreased hepatic *Fasn* mRNA levels in offspring.

Food restriction during pregnancy may directly influence the fetus through reduced nutrient availability, and indirectly through altered maternal metabolism. For this reason, we expected more pronounced changes in lipid metabolism in the F1 generation. However, such changes may manifest only under specific conditions (e.g., nutritional challenge) or at specific time points (e.g., later in life). It is worth mentioning that this study focused only on females, to verify whether the prenatal undernutrition can be directly transmitted to the next generation.

It is well known that DNA methylation controls the transcription of different genes. This process is environmentally flexible and can be altered by specific diets, drugs, age, disease, and other factors. The influence of different nutritional regimens on *Fasn* gene methylation has not yet been widely studied. In this study, methylation profile had no effect in changes in the liver as a response to a prenatal calorie-restricted diet. This suggests that the *Fasn* gene is not sensitive to environmental triggers transmitted through DNA methylation most likely due to the fact that the gene is transcribed mostly in the liver. On the other hand, Cordero [22] compared the effects of high-fat sucrose (HFS), HFS supplemented with methyl donors (HSFsupp), and control diets in adult rats, finding that the HFS diet increased body weight and changed plasma lipid profile; in the HFSsupp diet, however, these parameters were similar to those of the control animals. The hepatic mRNA level was only slightly induced as a response to methyl supplementation. Both test diets induced DNA methylation changes in the region spanning about 500 bp in the 5′-flanking region of the *Fasn* gene (from −688 to −1177). In our study, we examined the corresponding region and did not observe any changes in the DNA methylation profile in the calorie-restricted group. Moreover, most cytosines were completely unmethylated, reaching a maximum value for cytosine no. 2 in the F1 C group (16% average methylation). The methylation of the *Fasn* gene was also studied in adipose tissue. Gracia [23] studied the region close to TSS as well as CGIs located in a gene body. They used a high-fat/high-sucrose diet in adult rats and found that obesogenic feeding resulted in significant hypomethylation at the −90-bp cytosine position, as well as hypermethylation at the −62-bp cytosine position, according to TSS. It should also be noticed that DNA methylation is not the only epigenetic mechanism to affect gene expression patterns histone modifications also do so. This type of investigation for the *Fasn* gene was performed by Suzuki [24] in a rat model of insulin resistant animals. They found that the hepatic mRNA level of the *Fasn* gene in insulin-resistant rats was associated with increased methylation of lysine 4 in H3 histone, as well as with increased histone H3 and H4 acetylation. For this reason, it may be worth investigating whether the prenatally restricted diet can affect other epigenetic mechanisms, such as histone modifications, in terms of the *Fasn* gene.

## Conclusions

Calorie restriction during pregnancy leads to changes in *Fasn* mRNA levels in the liver and blood cholesterol concentrations in dams, but not in their four-week-old offspring. This may suggest that diet type may cause changes in gene expression profiles under specific physiological conditions, such as gestation, age, and others. The diet regimen investigated here did not alter hepatic DNA methylation of the *Fasn* 5′-flanking region in the rat, but other epigenetic mechanisms cannot be excluded as a regulators of *Fasn* gene expression.

## Additional files

Additional file 1: Figure S1. The 5′-flanking fragment of the *Fasn* gene examined in the methylation study (322 bp: blue bars represent primer sequences) overlapping CG dinucleotides (underlined by black bars). The transcription start site is marked with a red arrow. (JPEG 273 kb)

Additional file 2: Figure S2. Representative blots from Western Blot analyses: lines 1–3 represent samples of F0 C animals; lines 4–6 represent samples of F0 R animals. (JPEG 88 kb)

Additional file 3: ARRIVE checklist. (DOCX 657 kb)

## Abbreviations

C: Control group
CGI: CpG island
CO2: Carbon dioxide
HDL cholesterol: High-density lipoprotein cholesterol
IPTG: Isopropyl β-D-1-thiogalactopyranoside
IUGR: Intrauterine growth retardation syndrome
LDL cholesterol: Low-density lipoprotein cholesterol
R: Restricted group
RIPA: Buffer for protein extraction and immunoprecipitation
TBST: Tris-buffered saline with Tween 20 buffer
TG: Triglycerides
TSS: Transcription start site
X-Gal: 5-bromo-4-chloro-3-indolyl-β-D-galactopyranoside
