# Supplementary figures and images for: Prenatal caloric restriction alters lipid metabolism but not hepatic Fasn gene expression and methylation profiles in rats
Source: BMC Genet. 2017 Aug 15;18:78. doi: 10.1186/s12863-017-0544-0 (PMC5558693; doi:10.1186/s12863-017-0544-0)

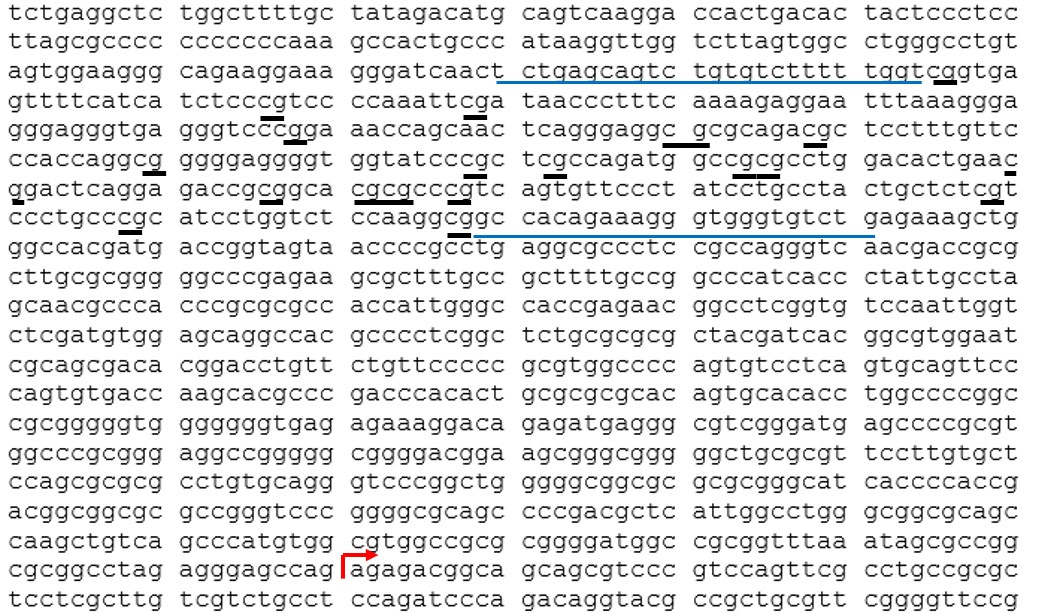

Supplement: Supplementary file 1 — The 5′-flanking fragment of the Fasn gene examined in the methylation study (322 bp: blue bars represent primer sequences) overlapping CG dinucleotides (underlined by black bars). The transcription start site is marked with a red arrow. (JPEG 273 kb) [file 12863_2017_544_MOESM1_ESM.jpg]

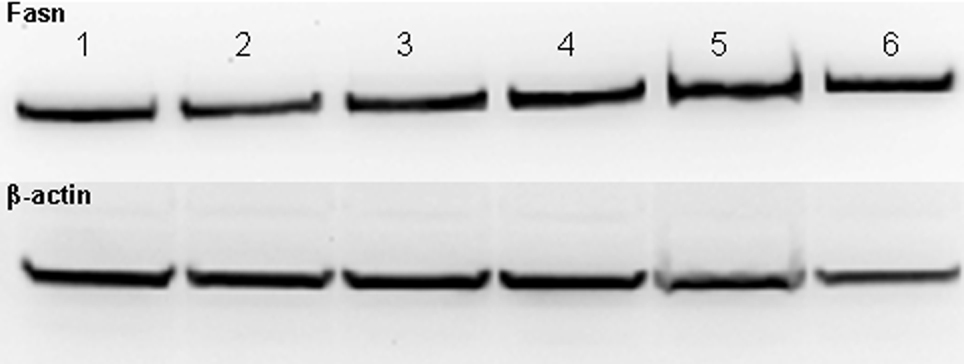

Supplement: Supplementary file 2 — Representative blots from Western Blot analyses: lines 1–3 represent samples of F0 C animals; lines 4–6 represent samples of F0 R animals. (JPEG 88 kb) [file 12863_2017_544_MOESM2_ESM.jpg]
