# Supplementary material for: Prenatal caloric restriction alters lipid metabolism but not hepatic Fasn gene expression and methylation profiles in rats
Source: BMC Genet. 2017 Aug 15;18:78. doi: 10.1186/s12863-017-0544-0 (PMC5558693; doi:10.1186/s12863-017-0544-0)

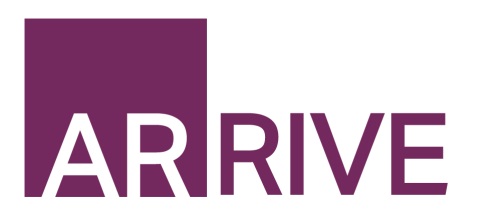


The ARRIVE Guidelines Checklist

Animal Research: Reporting In Vivo Experiments

Carol Kilkenny^1^, William J Browne^2^, Innes C Cuthill^3^, Michael Emerson^4^ and Douglas G Altman^5^

*^1^The National Centre for the Replacement, Refinement and Reduction of Animals in Research, London, UK, ^2^School of Veterinary Science, University of Bristol, Bristol, UK, ^3^School of Biological Sciences, University of Bristol, Bristol, UK, ^4^National Heart and Lung Institute, Imperial College London, UK, ^5^Centre for Statistics in Medicine, University of Oxford, Oxford, UK.*

|  | | ITEM | RECOMMENDATION | Section/ Paragraph |
| --- | --- | --- | --- | --- |
| 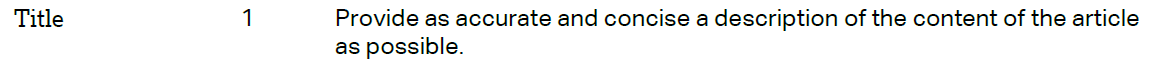 | | | Title |  |
| 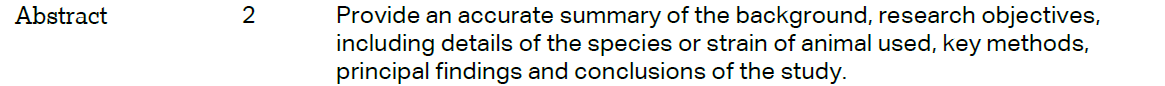 | | | Abstract |  |
| INTRODUCTION | | |  |  |
| 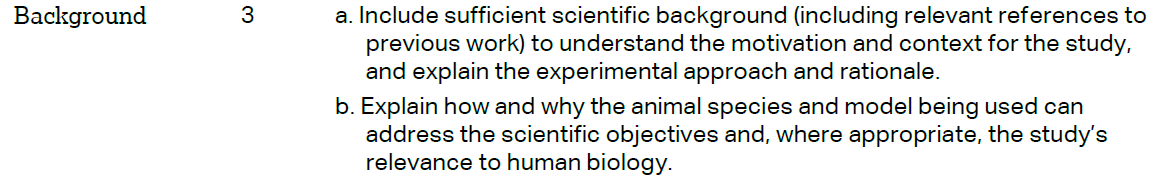 | | | Background paragraphs 1-3  Background  paragraph 1 |  |
| 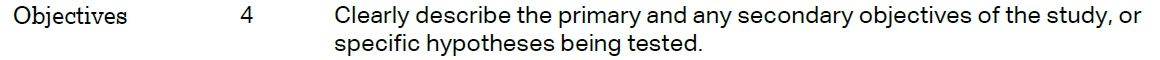 | | | Background paragraph 4 |  |
| METHODS | | |  |  |
| 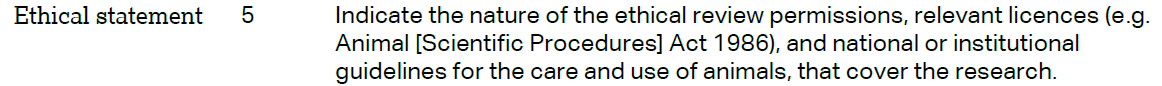 | | | Material and methods; part 1 |  |
| 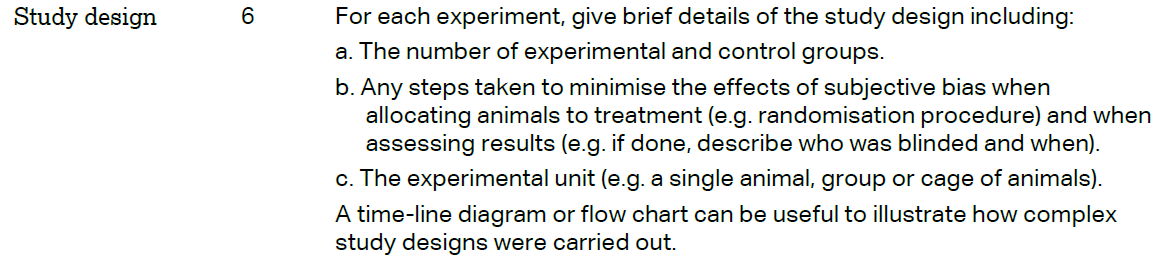 | | | Material and methods; part 1 |  |
| 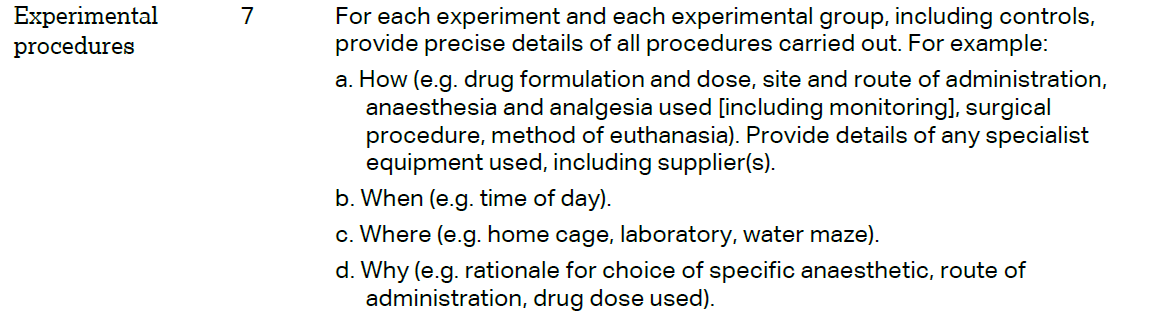 | | | Material and methods; parts 1-5 |  |
| 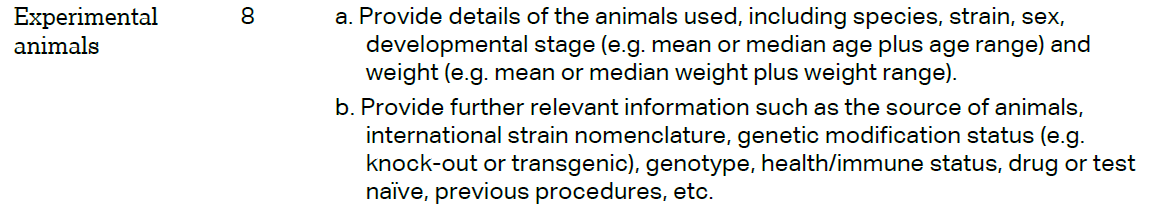 | | | Material and methods; part 1 |  |

The ARRIVE guidelines. Originally published in *PLoS Biology*, June 2010^1^

| 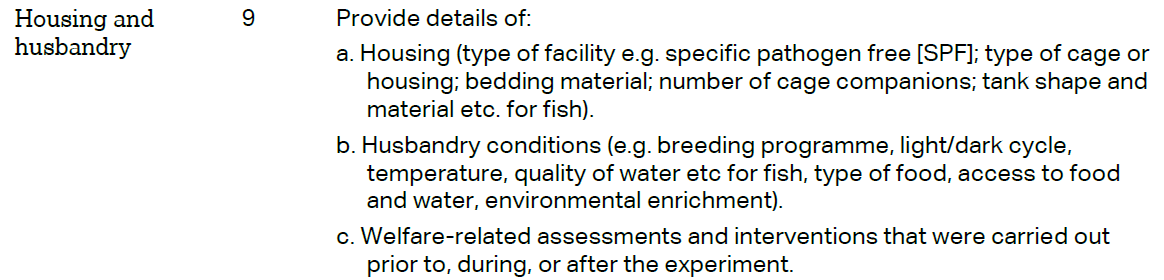 | Material and methods; part 1 | |
| --- | --- | --- |
| 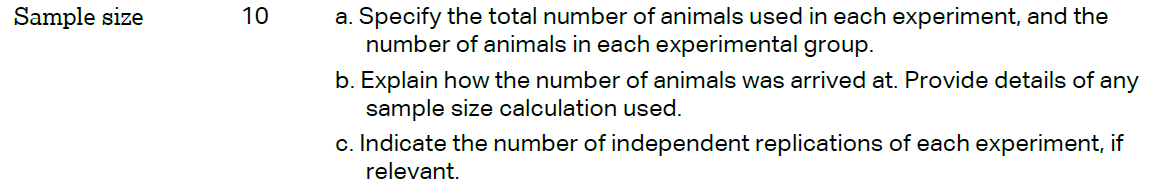 | Material and methods; parts 1-5 | |
| 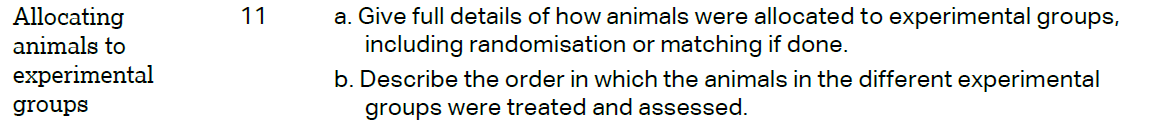 | Material and methods; part 1 | |
| 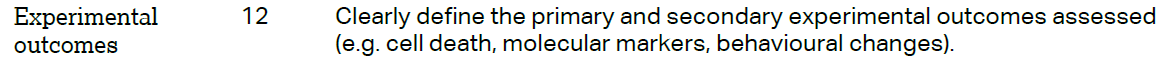 | Material and methods; parts 2-5 | |
| 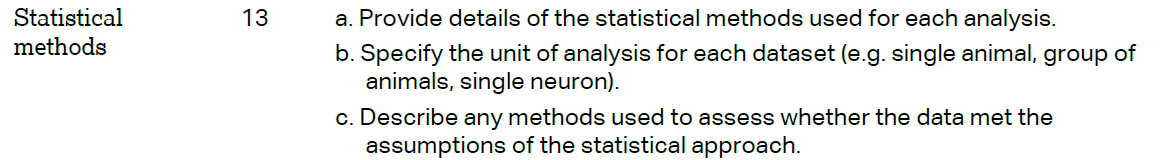 | Material and methods; part 6 | |
| RESULTS |  | |
| 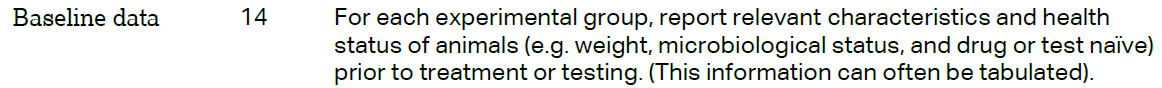 | Results; part 1 | |
| 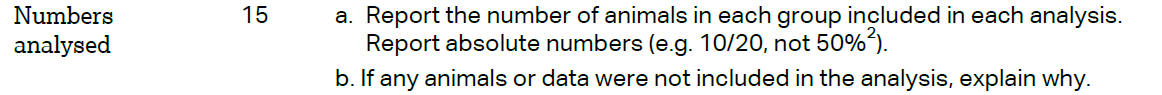 | Material and methods; part 1 | |
| 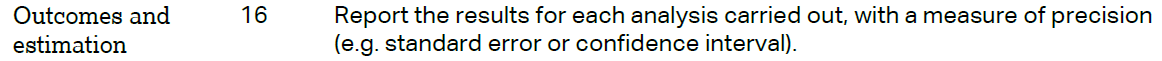 | Results parts 1-3 and figures 1-4 | |
| 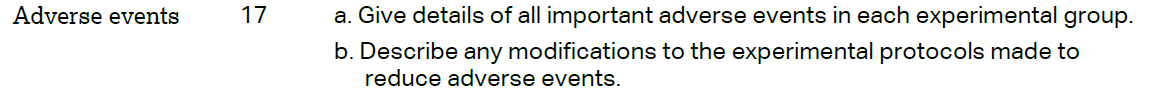 | Material and methods; parts 2-6 | |
| DISCUSSION |  | |
| 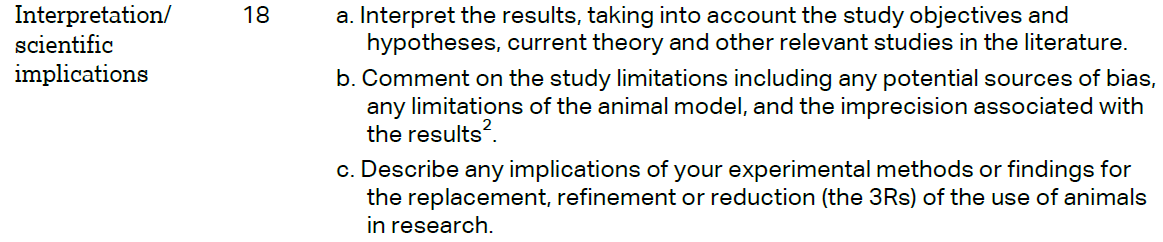 | Discussion, paragraphs1-3 | |
| 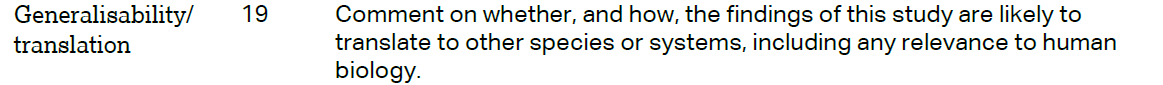 | Conclusion paragraph 1 | |
| 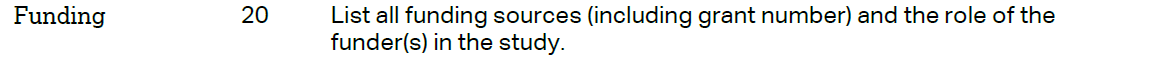 | | Declarations, founding section |


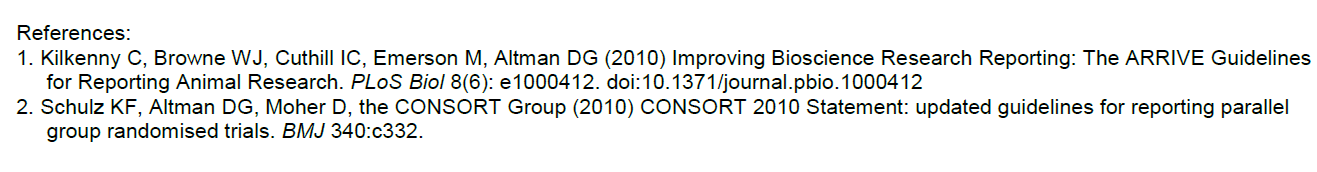

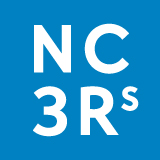

Supplement: Supplementary file 3 — ARRIVE checklist. (DOCX 657 kb) [file 12863_2017_544_MOESM3_ESM.docx]
